# Hemodynamic protective effects of epinephrine containing saline irrigation in biportal endoscopic lumbar surgery

**DOI:** 10.1097/MD.0000000000029311

**Published:** 2022-07-29

**Authors:** Woo-Hyeong Ko, Yong-Hyun Cho, Won Jang, Sun-Hee Kim, Hyun-Seok Lee, Hyun-Cheol Ko, Jae-Hyun Kwon

**Affiliations:** Department of Anesthesiology and Pain Medicine, Seoul Sungsim General Hospital, Seoul, Korea.

**Keywords:** biportal endoscopic spine surgery, epinephrine, hypotension, irrigation, retrospective observational study

## Abstract

During endoscopic orthopedic surgery, epinephrine mixed with irrigation saline is frequently used to improve visualization. By monitoring hemodynamic parameters throughout the procedure, we intended to discover the hemodynamic effect of epinephrine between the normal saline irrigation fluid without epinephrine group (NS) and normal saline irrigation fluid with epinephrine group (EPI).

Patients who underwent 1-level lumbar decompression or discectomy surgery without fusion between August 2019 and July 2020 were reviewed retrospectively. The hemodynamic parameters were compared between the NS group and EPI group. As a second endpoint, the incidence of hypotension and hypertension events, expected blood loss, postoperative nausea and vomiting and postoperative epidural hematoma were compared between the 2 groups.

The 2 groups were homogeneous in terms of age, sex, weight, height, body mass index (BMI), ASA physical status (ASA PS), and diagnosis. The incidence of hypotension events (67.2 % in the NS group, 45.7 % in the EPI group, *P* =.015) and severe hypotension events (51.7 % in the NS group, 28.6 % in the EPI group, *P* = .015) were less frequent in the EPI group. Only epinephrine had a significant protective effect through a multivariable analysis (*P* = .027, OR = 2.361) and in severe hypotension events, only epinephrine had a significant protective effect through a multivariable analysis (*P* = .011, OR = 2.818), and EBL was the risk factor through a multivariable analysis (*P* = .016, OR = 1.002)

We believe that the addition of epinephrine to irrigation saline has hemodynamic protective effects in patients who underwent endoscopic lumbar surgery.

## 1. Introduction

In knee and shoulder arthroscopic surgery, epinephrine is frequently added to the irrigation fluid to reduce intraoperative articular bleeding, thereby improving visualization.^[1–3]^ Recently, in routine arthroscopic knee surgery, the addition of epinephrine to saline irrigation fluid at a concentration of 1 mg/L significantly reduced the need for the use of a tourniquet.^[1,4]^ However, there are some case reports of severe refractory hypertension and lethal arrhythmia when using epinephrine mixed saline irrigation fluid in shoulder arthroscopic surgery.^[5,6]^ In our institute, while performing biportal endoscopic spine surgery (BESS), saline irrigation pressure makes the working space similar to a joint arthroscopic surgery, and 1 mg of epinephrine is added to 3 L of normal saline irrigation fluid, as in knee and shoulder surgery.^[4]^ We also used a saline infusion pump with a pressure of 50 mm Hg, which is higher than the average venous pressure of 40 mm Hg to prevent venous bleeding for visualization.^[4]^

When using neuraxial techniques such as epidural anesthesia, to provide better analgesia/anesthesia results, opioids and nonopioid drugs are commonly administered intrathecally or epidurally in conjunction with local anesthetics.^[7]^ When a small amount of epinephrine is injected peridurally, the slowly absorbed epinephrine produces a predominant β-adrenergic stimulation, which causes an increase in HR, SV, and CO and a decrease in TPR, resulting in a decrease in MAP. This is because only β-adrenergic receptors respond to low concentrations of epinephrine.^[7–9]^ However, if a sufficient amount of epinephrine is absorbed, vasoconstriction will occur under the influence of α-adrenergic receptors, leading to an increase in blood pressure.

The epidural space is a potential space and the volume of it is not small, considering it takes about 1.5–2.0 ml of a local anesthetic to block a spinal segment in the epidural space,^[10,11]^ and the epidural injection of 40 ml of lidocaine with radiocontrast agent resulted in extensive spreads ascending to the upper thoracic and even cervical levels.^[12]^ Increased intracranial pressure during endoscopic lumbar surgery due to increased epidural pressure caused by irrigation pressure means that there is an amount of irrigation solution with epinephrine during surgery in the epidural space.^[13]^

In our institute, we used at least 10 mg of epinephrine as an additive to the irrigation solution. Thus, it can be assumed that a significant amount of epinephrine is present in the epidural space and even in the systemic circulation, and its effect manifests in hemodynamic changes. The purpose of this retrospective study between 2 similar groups was to determine whether the use of saline irrigation with epinephrine during endoscopic spine surgery would significantly affect hemodynamic values when compared with the use of normal saline without epinephrine irrigation fluid. We compared the hemodynamic changes in the group using normal saline irrigation with epinephrine and the group using normal saline irrigation without epinephrine in endoscopic spine surgery, and also investigated the factors that cause these hemodynamic changes.

## 2. Methods

This study received approval from the public institutional review board (PO1-202107-21-001), and written informed consent from the patients was waived. This was a retrospective cohort study in which we reviewed the medical records including anesthesia record of patients who received a 1-level lumbar decompression or discectomy surgery without fusion between August 2019 and July 2020. Before February 2020, all patients received BESS with normal saline irrigation fluid without epinephrine (NS group). Thereafter, we treated all patients with BESS with normal saline irrigation fluid with epinephrine (EPI group).

### 2.1. Patients; inclusion and exclusion criteria

A sample of 212 patients who underwent BESS and who were under the care of 2 staff orthopedic surgeons at our institution during the study period were analyzed. A total of 112 patients were treated with normal saline irrigation, and 100 patients were treated with normal saline irrigation fluid with epinephrine. We subsequently excluded patients who underwent spinal anesthesia, who underwent surgery for more than 1 segment, who had ASA physical status III or higher, who were <18 or >80 years of age at that time. Progress notes were reviewed for postoperative complications. We excluded patients with dural tears as a postoperative complication because of the possibility of epinephrine absorption into the intrathecal space.

### 2.2. Anesthesia methods

Anesthesia was induced with 2.0 mg/kg propofol and 0.1 μg/kg/min remifentanil and maintained with 1.0 to 2.5 vol.% isoflurane and nitrous oxide with 0.1 to 0.5 μg/kg/min remifentanil. Rocuronium was administered as a neuromuscular blocking agent. Ephedrine and phenylephrine were used as vasopressors, and nicardipine and esmolol were used as antihypertensive agents.^[14–16]^ Ondansetron was administered at the end of surgery for PONV prophylaxis in all patients.^[17,18]^

### 2.3. Data acquisition

For every case, the investigator reviewed the anesthesia records. Demographic data, including age, ASA physical status classification (ASA PS), sex, and weight were also extracted from the anesthesia records. Preoperative systolic blood pressure (SBP) and diastolic blood pressure (DBP) at the time of admission by oscillometric BP cuffs, and heart rate (HR) recordings were obtained from medical records as basal values. Intraoperative BP measured at 5-minute intervals from oscillometric BP cuffs or intraarterial line and transducer systems were obtained from scanned anesthesia records. After the surgical incision, not after anesthesia induction, the lowest and highest blood pressure and heart rate at that time were recorded, and the use of vasopressors and antihypertensive drugs was checked. The Koivuranta postoperative nausea and vomiting (PONV) risk factor score and PONV events were analyzed from the medical records.^[17,18]^ The Koivuranta PONV score is based on 5 predictors: female sex, history of PONV and/or motion sickness, nonsmoking status, use of postoperative opioids length of surgery >60 minutes.^[17,18]^ All mean arterial pressure (MAP) values were calculated using the formula: [(2 × diastolic BP (DBP)) +systolic BP (SBP)]/3. All estimated blood loss (EBL) values were calculated using the formula: [estimated blood volume (EBV) × {initial hematocrit (Hcti) – final hematocrit (Hctf) + transfused RBC volume}]/mean hematocrit.^[19]^ We measured the postoperative hemodynamic parameters for 1 week after surgery.

### 2.4. Operation methods

In BESS, in the prone position, 2 unilateral portals (1 for endoscopy and the other for instrumental working) were made.^[4]^ We used a saline infusion pump with a pressure of 50 mm Hg, which is higher than the average venous pressure of 40 mm Hg to prevent venous bleeding.^[4]^ A vacuum suction drain was placed and connected to a negative pressure bag of 120 ± 30 mm Hg in all cases, and it was removed on day 2 after the operation.^[4]^

### 2.5. Definition of arterial hypotension and hypertension

There is a progressive increased association for each absolute MAP threshold (≤ 65, ≤ 55) for major adverse cardiac or cerebrovascular events (MACCE) and 30- and 90-day mortality.^[20]^ Under the 40% below baseline relative MAP threshold, intraoperative hypotension was associated with MACCE.^[20]^ Based on this, a hypotension event was defined as at least 1 measurement of MAP ≤ 65 mm Hg or at least 1 measurement 40% lower than the baseline or incidence of ephedrine or phenylephrine infusion. Severe arterial hypotension was defined as at least 1 measurement of MAP ≤ 55 mm Hg or at least 1 measurement 40% lower than the baseline or incidence of ephedrine or phenylephrine infusion. Arterial hypertension was defined as at least 1 measurement of SBP 140 mm Hg or at least 1 measurement 40% higher than the baseline or the incidence of nicardipine or esmolol infusion.^[21]^

### 2.6. Statistical analysis

Parametric variables were analyzed with Student t-test, and nonparametric variables were analyzed using Fisher exact test and chi-square test. ASA classification and Koivuranta PONV risk factor scores were compared using the Mann–Whitney *U* test. The protective and risk factors for hypotension or hypertension were analyzed using a multivariable logistic regression test. SPSS for Windows (ver. 16.0; SPSS Inc., Chicago, IL, USA) was used.

## 3. Results

After reviewing the medical records and anesthesia records, 128 out of 212 patients were eligible for inclusion: 58 in the normal saline group (NS) and 70 in the epinephrine mixed saline group (EPI). The 2 groups were homogenous in age, sex, weight, height, BMI, ASA PS, including hypertension (HTN) and diabetes (DM), and diagnosis (Table 1).

**Table 1:** Demographic data.

Variable	NS* (n = 58)	EPI† (n = 70)	*P* value
Age	65.93 ± 11.17	66.50 ± 10.35	.766
Sex (F/M)‡	32/26	32/38	.287
Height (cm)	159.43 ± 8.81	161.26 ± 9.23	.257
Weight (kg)	65.10 ± 10.92	67.06 ± 10.92	.318
BMI§ (kg/m^2^)	25.53 ± 3.16	25.74 ± 3.43	.720
ASA PS∥ (I/II)	14/44	18/52	.838
Hypertension	36 (62.1 %)	32 (45.7%)	.065
Diabetes	14 (24.1 %)	32 (25.7%)	.838
Diagnosis SS¶/HNP**/spondylolisthesis/ASD††	32/16/7/3	32/19/13/6	.578

Basal hemodynamic values were not significantly different. The intraoperative lowest SBP of the NS and EPI groups were 91.03 mm Hg and 94.64 mm Hg, respectively. The difference was significant (*P* =.010) (Table 2). The intraoperative highest SBP of NS and EPI groups were 108.62 mm Hg and 112.86 mm Hg, respectively. The difference was significant (p =.018) (Table 2). The incidence of hypotension was significantly less frequent in the EPI group (45.7 %) than in the NS group (67.2 %) (*P* =.015) and severe hypotension events were also less frequent in the EPI group (28.6 %) than in the NS group (51.7 %) (*P* =.008) (Table 3).

**Table 2:** Hemodynamic parameters.

Variable	NS* (n = 58)	EPI† (n = 70)	*P* value
Basal values			
SBP‡	126.33 ± 8.82	128.49 ± 8.83	.171
DBP§	76.33 ± 8.18	77.06 ± 7.92	.610
MAP∥	92.99 ± 7.20	94.20 ± 7.22	.348
HR¶	72.69 ± 9.21	71.51 ± 9.26	.475
Intraoperative lowest values			
SBP‡	91.03 ± 6.12	94.64 ± 8.94	.010**
DBP§	53.02 ± 7.49	54.00 ± 6.46	.434
MAP∥	65.69 ± 6.55	67.55 ± 6.70	.117
HR¶	62.81 ± 8.66	65.17 ± 8.36	.120
Intraoperative highest values			
SBP‡	108.62 ± 8.37	112.86 ± 11.05	.018**
DBP§	62.24 ± 9.47	60.93 ± 9.10	.427
MAP∥	77.70 ± 8.07	78.24 ± 9.07	.727
HR¶	65.95 ± 8.53	67.26 ± 7.49	.357
Postoperative lowest values			
SBP‡	105.43 ± 10.61	106.90 ± 9.94	.421
DBP§	60.59 ± 9.75	62.06 ± 10.49	.416
MAP∥	75.53 ± 8.83	77.00 ± 9.04	.357
HR¶	71.91 ± 7.71	70.41 ± 10.00	.352
Postoperative highest values			
SBP‡	149.41 ± 13.41	149.80 ± 12.60	.867
DBP§	84.138 ± 9.57	82.34 ± 9.36	.287
MAP∥	105.90 ± 8.33	104.83 ± 8.27	.470
HR¶	74.45 ± 11.49	75.29 ± 13.90	.715

**Table 3:** Hemodynamic event.

Variable	NS* (n = 58)	EPI† (n = 70)	*P* value
Hypotension event‡	39 (67.2%)	32 (45.7%)	.015¶
Severe hypotension event§	30 (51.7%)	20 (28.6%)	.008**
Hypertension event∥	0 (0.0%)	4 (5.7%)	.126

The operative time did not show a significant difference (Table 4). There were no significant differences in the intraoperative bleeding (Table 4). The mean Koivuranta PONV score in NS group was 2.52 ± 0.63 and in EPI group was 2.49 ± 0.76. The difference was not significant (*P* = .816). The PONV events did not show a significant difference (2 events in NS group and 4 events in EPI group, *P* = .689).

**Table 4:** Operative time and Intraoperative bleeding

Variable	NS* (n = 58)	EPI† (n = 70)	*P*-value
Operative time (min)	83.97 ± 23.39	85.14 ± 23.14	.776
Hbi‡	13.67 ± 1.37	13.87 ± 1.74	.477
Hcti§	40.94 ± 4.11	41.03 ± 5.31	.913
Hbf∥	12.53 ± 1.41	12.77 ± 12.77	.369
Hctf¶	37.63 ± 4.23	37.67 ± 4.63	.956
EBL**	375.52 ± 239.06	394.05 ± 202.85	.636

The protective and risk factors of hypotension events and severe hypotension events were analyzed in all subjects. In the hypotension event, only epinephrine had a significant protective effect through a multivariable analysis (*P* = .027, OR = 2.361) (Table 5). In severe hypotension events, only epinephrine had a significant protective effect through a multivariable analysis (*P* = .011, OR = 2.818), and EBL was a risk factor in the multivariable analysis (*P* = .016, OR = 1.002) (Table 6).

**Table 5:** Risk factors for Hypotension event*, univariable and multivariable

			*P* value	
Hypotension event*	+(71)	- (57)	Univariable	Multivariable
Epinephrine (EPI/NS)†	32/39	38/19	.016∥	.027∥ OR¶:2.361
Age	68.10 ± 7.55	63.93 ± 13.34	.036∥	.125
Sex (F/M)‡	30/41	23/34	.052	.172
HTN(+/-)	43/28	25/32	.061	.319
DM(+/-)	16/55	16/41	.472	
Operative time (min)	85.77 ± 19.62	83.16 ± 27.07	.525	
EBL§	390.52 ± 234.56	379.59 ± 200.55	.778	

**Table 6:** Risk factors for Severe hypotension event*, univariable and multivariable

			*P* value	
Severe hypotension event*	+(50)	- (78)	Univariable	Multivariable
Epinephrine (EPI/NS)†	50/30	28/20	0.008¶	0.011¶ OR**: 2.818
Age	69.04 ± 7.38	64.45 ± 12.06	0.022∥	0.125
Sex (F/M)‡	29/21	35/43	0.149	
Hypertension (+/-)	34/16	34/44	0.008¶	0.086
Diabetes (+/-)	9/41	23/55	0.147	
Operative time (min)	86.80 ± 21.23	83.21 ± 24.36	0.393	
EBL§ (ml)	449.99 ± 230.88	344.41 ± 202.44	0.010∥	0.016∥ OR**:1.002

The incidence of postoperative epidural hematoma between the 2 groups (0.00 % in NS group and 7.14 % in EPI group) was close to the significant difference (*P* = .063).

## 4. Discussion

In knee and shoulder arthroscopy, epinephrine is frequently added to the irrigation fluid to reduce intraoperative articular bleeding, thereby improving visualization.^[1–3]^ The results of the previous study indicate that epinephrine irrigation fluid compared with standard saline irrigation has potential benefits by reducing intraarticular bleeding, and no hemodynamic changes were observed.^[1–3]^ However, in spine endoscopic surgery, there has been no study on securing the visual field with epinephrine, and there have been no studies on hemodynamic changes due to epinephrine. Moreover, unlike other intraarticular surgeries, in endoscopic spine surgery, the soft tissue is continuously exposed to irrigation solution, the volume of the epidural space is expected to be significant, and the irrigation solution usage is approximately 30 L or more, which is higher than that for arthroscopic surgery; therefore, it is thought that the amount of systemic absorption of epinephrine in spine surgery is greater than that of arthroscopic surgery.^[4,10–12]^ Based on the above factors, we assumed that there were differences in hemodynamic parameters.

In this study, hemodynamic changes that occurred after surgical incision in the EPI and NS groups were investigated in patients who underwent endoscopic spinal surgery under general anesthesia. The lowest blood pressures were observed during surgery, and SBP, DBP, MAP, and HR were all higher in the EPI group, but SBP was only statistically significant. In the case of the highest intraoperative blood pressure, only SBP was significantly higher. Considering that the 2 groups are demographically homogeneous, this result can be attributed to the action of adrenergic receptors by epinephrine. β2-adrenergic receptors are known to lower blood pressure by causing vasodilation in response to relatively low concentrations of epinephrine.^[7–9]^ However, considering the higher blood pressure and HR in the EPI group, it can be thought that vasoconstriction and myocardial contractility increased due to the α1- and β1-adrenergic effects because epinephrine was maintained at a relatively high concentration during spine surgery.

The incidence of hypotension events (67.2 % in the NS group, 45.7 % in the EPI group, *P* = .015) and severe hypotension events (51.7 % in the NS group, 28.6 % in the EPI group, *P* = .015) defined in this study were less frequent in the EPI group. In spine surgery, controlled or deliberate hypotension is commonly used to reduce intraoperative bleeding to improve surgical visualization and result in faster surgeries and, thus, further reduce transfusion dependence.^[22]^ However, intraoperative hypotension during noncardiac surgery is common and is associated with increased major adverse cardiac or cerebrovascular events and acute kidney injury.^[20]^ Therefore, it is important to maintain hemodynamic parameters to prevent hypotension during surgery. In this regard, it can be said that using normal saline irrigation containing epinephrine during surgery has hemodynamic advantages over normal saline irrigation. In addition, this benefit has a statistically strong association with severe hypotension, which may cause more adverse cardiac or cerebrovascular events or AKI after surgery.^[20]^ Therefore, the use of epinephrine can be considered as a hemodynamic protective factor for hypotension and severe hypotension events. In addition, extensive blood loss was found to be a risk factor for severe hypotension events.

On the other hand, intraoperative hypertension can be considered as an unwanted reaction that can occur when epinephrine is absorbed systemically. There have also been several case reports in which patients were at risk due to severe refractory hypertension or arrhythmia during shoulder arthroscopic surgery with irrigation containing epinephrine.^[5,6]^ However, in this study, the difference in hypertension events between the 2 groups was not statistically significant, and there were no patients with severe hemodynamic consequences. It can be considered that epinephrine added irrigation in spine endoscopic surgery may not pose a great risk.

There have been no studies on whether epinephrine-added irrigation in spine endoscopic surgery actually provides a better visual field for the operator. Although the operator’s visual field could not be assessed directly in this study because patients were not randomized, the 2 orthopedic surgeons who performed spine endoscopic surgery in this study noted that the use of epinephrine improved visualization. However, as indirect indicators of visual field, the expected blood loss using pre- and postoperative hematocrit, body weight, and operative time showed no statistically significant difference. This may mean that bleeding, which has a significant effect on the visual field during surgery, may not differ significantly. However, although not statistically significant, considering the fact that postoperative epidural hematomas were more common in the epinephrine group, it can be assumed that intraoperative bleeding was less in the EPI group, and more postoperative bleeding in the unhemostasised vessels may have formed hematomas. However, since studies have shown that epinephrine injection reduces bleeding after surgery, this idea is contrary to existing studies, so further research is needed.^[23,24]^

Although the mechanisms of epinephrine-induced nausea and vomiting have not been clearly elucidated, it is likely through the central effects of α-adrenergic receptor stimulation.^[7,25,26]^ In this study, the Koivuranta PONV risk factor scores between the EPI and NS groups were relatively consistent, and there was no significant difference in the PONV event between the 2 groups, and the hemodynamic parameters after surgery were not significantly different between the 2 groups.^[17,18]^ Therefore, it is thought that the level of circulatory epinephrine after surgery is unlikely to affect the incidence of nausea and vomiting.

There were some limitations to this study. In this study, we could not directly determine whether the absorption of epinephrine in the body actually occurred, because the serum epinephrine concentration change during surgery was not measured. Because this was a retrospective study, the 2 groups were not completely homogenous. However, we were able to set both groups to be almost homogeneous by applying exclusion criteria such as ASA PS, and the risk factor analysis to all subjects with multivariable analysis would offset the drawback. The sample size was not sufficient to verify statistical significance for some of our assumptions. In addition, since the present study concluded that the use of epinephrine in spinal endoscopic surgery does not pose a significant risk to patients, it may be possible to randomize patients in a follow-up study of the effect of epinephrine on intraoperative visual field.

## 5. Conclusions

The incidences of hypotension and severe hypotension were lower in the epinephrine group. We believe that the addition of epinephrine to irrigation saline has hemodynamic protective effects in patients who undergo endoscopic lumbar surgery. There were no differences in hypertension events as a side effect of epinephrine and no differences in postoperative complications when using epinephrine.

## Author contributions

Conceptualization: Woo-Hyeong Ko, Yong-Hyun Cho.Formal analysis : Won Jang.Methodology : Sun-Hee Kim, Hyun-Seok Lee. Investigation : Hyun-Cheol Ko, Jae-Hyun Kwon.Writing – original draft : Woo-Hyeong Ko.Writing – review & editing : Woo-Hyeong Ko, Yong-Hyun Cho.

## Footnotes

* NS = Normal saline irrigation group.

† EPI = Epinephrine mixed normal saline irrigation group.

‡ F/M = Female/Male.

§ BMI= Body mass index.

∥ ASA PS = The American Society of Anesthesiologists physical status.

¶ SS = Spinal stenosis.

** HNP = Herniated nucleus pulposus.

†† ASD = Adjacent segmenetal disease.

* NS = Normal saline irrigation group.

† EPI = Epinephrine mixed normal saline irrigation group.

‡ SBP = Systolic blood pressure (mm Hg).

§ DBP = Diastolic blood pressure (mm Hg).

∥ MAP = Mean arterial pressure (mm Hg).

¶ HR = Heart rate (per min).

** *P* value of <.05.

* NS = Normal saline irrigation group.

† EPI = Epinephrine mixed normal saline irrigation group.

‡ Hypotension event= At least 1 measurement of MAP ≤ 65 mm Hg or at least 1 measurement 40% lower than the baseline or incidence of ephedrine or phenylephrine infusion.

§ Severe hypotension event= At least 1 measurement of MAP ≤ 55 mm Hg or at least 1 measurement 40% lower than the baseline or incidence of ephedrine or phenylephrine infusion.

∥ Hypertension event= At least 1 measurement of SBP 140 mm Hg or at least 1 measurement 40% higher than the baseline or the incidence of nicardipine or esmolol infusion.

¶ *P* value of <.05.

**
*P* value of <.01.

* NS = normal saline irrigation group.

† EPI = epinephrine mixed normal saline irrigation group.

‡ Hbi = initial hemoglobin.

§ Hcti = initial hematocrit.

∥ Hbf = final hemoglobin.

¶ Hctf = final hematocrit.

** EBL = estimated blood loss.

* Hypotension event = at least 1 measurement of MAP ≤ 65 mm Hg or at least 1 measurement 40% lower than the baseline or incidence of ephedrine or phenylephrine infusion.

† EPI/NS = normal saline irrigation group/epinephrine mixed normal saline irrigation group.

‡ F/M = female/male.

§ EBL = estimated blood loss.

∥ *P* value of <.05.

¶ OR = Odds ratio.

* Severe hypotension event = at least 1 measurement of MAP ≤ 55 mm Hg or at least 1 measurement 40% lower than the baseline or incidence of ephedrine or phenylephrine infusion.

† EPI/NS = normal saline irrigation group/epinephrine mixed normal saline irrigation group.

‡ F/M = female/male.

§ EBL = estimated blood loss.

∥ *P* value of <.05.

¶ *P*-value of <.01.

** OR = odds ratio.
